# Beyond simple small-angle X-ray scattering: developments in online complementary techniques and sample environments

**DOI:** 10.1107/S2052252514019198

**Published:** 2014-09-23

**Authors:** Wim Bras, Satoshi Koizumi, Nicholas J Terrill

**Affiliations:** a Netherlands Organization for Scientific Research (NWO), DUBBLE@ESRF, BP 220, 6 Rue Jules Horowitz, Grenoble 38043, France; b College of Engineering, Ibaraki University, 1-1 Namiki, Tsukuba, Ibaraki 305-0044, Japan; c Science Division, Diamond Light Source, Harwell Science and Innovation Campus, Didcot, Oxfordshire OX11 0DE, UK

**Keywords:** SAXS, WAXS, SANS, complementary techniques, sample environment

## Abstract

Small- and wide-angle X-ray scattering (SAXS, WAXS) are standard tools in materials research. The simultaneous measurement of SAXS and WAXS data in time-resolved studies has gained popularity due to the complementary information obtained. Furthermore, the combination of these data with non X-ray based techniques, *via* either simultaneous or independent measurements, has advanced understanding of the driving forces that lead to the structures and morphologies of materials, which in turn give rise to their properties. The simultaneous measurement of different data regimes and types, using either X-rays or neutrons, and the desire to control parameters that initiate and control structural changes have led to greater demands on sample environments. Examples of developments in technique combinations and sample environment design are discussed, together with a brief speculation about promising future developments.

## Abstract 2

Possibilities in auxiliary technique combinations with small- and wide-angle X ray scattering are described, as well as more complicated sample environments used in X-ray and neutron scattering.

## 1. Introduction   

When hard X-ray synchrotron radiation first became available, time-resolved SAXS was one of the techniques that attracted several pioneers. The earliest SAXS beamline was constructed in Hamburg, in order to elucidate the mechanism by which muscles contract (Rosenbaum *et al.*, 1971 ▶). Somewhat later, when the beamlines and detectors (Gabriel *et al.*, 1978 ▶) had improved significantly, it became possible to follow dynamically the effects of, for example, heating and cooling in polymers, by simultaneously monitoring scattering at small and wide angles – the latter potentially including diffraction peaks arising from order in the material. This allowed questions to be asked about the role that hierarchical length scales play in the physical properties of the materials under study (Bark *et al.*, 1992 ▶). The early experiments were carried out in a rather improvised fashion but were soon followed by the construction of the first dedicated SAXS/WAXS beamline at the Daresbury synchrotron (Bras *et al.*, 1993 ▶). The nearly parallel developments in Hamburg and Daresbury indicate that the time was ready for this. Indeed, since those early days the majority of new SAXS beamlines, with the exception of those dedicated to protein solution scattering, have been equipped with a WAXS option, making it impracticable to compile a comprehensive list (Riekel *et al.*, 1996 ▶; Bras *et al.*, 2003 ▶; Kellermann *et al.*, 1997 ▶; Kirby *et al.*, 2013 ▶; Hexemer *et al.*, 2010 ▶).

A conventional SAXS/WAXS beamline has two detectors placed several metres apart to collect SAXS and WAXS data simultaneously. With the lower divergence of third-generation synchrotron X-ray sources, the compromise between under- and over-focusing of the beam between the two detectors is reduced to an acceptable level. While high collimation allows the collection of both SAXS and WAXS data on a single detector, this arrangement will be detrimental to the low-angle information content, where a very large *q* range [*q* = (4π/λ)sinθ, where θ is half the scattering angle and λ is the wavelength of the incident radiation] would be projected onto a limited number of detector pixels.

For some time, the photon flux delivered by most beamlines exceeded the capabilities of the detectors available. However, with the introduction of efficient high count-rate semiconductor-based photon-counting detectors (Schmitt *et al.*, 2004 ▶; Broennimann *et al.*, 2006 ▶), a threshold was passed beyond which one could be less concerned about the quality of the beam and detectors. As a result, it was possible to focus increasingly on the development of sample environments, data analysis methodology and further combinations with non X-ray based techniques. These last were already well under way in the 1990s (Bras & Ryan, 1998 ▶) and they have since been facilitated by technical developments in other areas, such as IR and UV fibre optics required for Raman scattering and FT–IR. The shift in development to more complicated sample environments has facilitated the design of experiments in which specific sample conditions can be controlled or perturbed. Industrial processing conditions, for example, can be mimicked (Portale *et al.*, 2013 ▶), and models of working devices studied during their operating cycle (Gleeson *et al.*, 1995 ▶; Siemianowski *et al.*, 2012 ▶) can be incorporated into the data collection.

It is not practicable to provide here a comprehensive overview of the developments in online time-resolved techniques used in combination with SAXS/WAXS (‘hyphenated’ techniques) and the accompanying more complicated sample environments. Instead, we try to give a flavour of some of the developments, with an emphasis on materials science experiments. Where possible, hints as to the timeline of how certain areas have developed in the last 20 years are given.

## 2. Which time resolutions, when to use and when to be careful?   

The combination of techniques is not always beneficial. If the timescale over which samples are modified is such that it is also feasible to perform independent experiments without introducing a notable error margin between the experiments, then one is better off carrying out optimized independent experiments. In combinations, often at least one of the experiments yields suboptimal, but still useable, data. The only advantage of combining techniques is the synergy that results when data sets are obtained simultaneously from a single sample with, for example, a well characterized thermal history or other provenance. The longer limit we define, somewhat arbitrarily, as being 1–2 min per time frame. The limit on the fast side is in general no longer set by the available X-ray flux or the detectors. Where the fastest of the older generation of CCD area detectors could collect data at rates of 15 Hz with a limited dynamic range (Labiche *et al.*, 2007 ▶), the more modern commercially available photon-counting Pilatus detectors (Kraft *et al.*, 2009 ▶) can achieve rates of up to 500 Hz with a greatly increased dynamic range. These limits will be pushed further by the detector manufacturers in the coming years (Dinapoli *et al.*, 2011 ▶; Radicci *et al.*, 2012 ▶). The main limitation has now become the speed with which samples can be perturbed homogeneously. This rate depends, to a large extent, on what kinds of materials are being investigated. A very fast, but controlled and gradient-free, thermal quench would be easy for metal foils but more difficult for thermally insulating materials (Cavallo *et al.*, 2010 ▶). Other examples are chemical or physical reactions that require the mixing of two liquids (Angelov *et al.*, 2013 ▶). Here, the limiting step in obtaining information about the structures formed during a chemical reaction would not be the difference in electron density between incompletely mixed fluids, but rather the time required to obtain a completely homogeneous liquid (Laidler, 1987 ▶).

Very fast reactions can be studied using microfocus techniques, where a miniature extruder, *e.g.* a spider (Riekel & Vollrath, 2001 ▶), or colliding microbubbles (Akiyama *et al.*, 2002 ▶) are used. The distance between the point where the reaction is initiated (*i.e.* the spinnerets of the spider or the point of collision between two microbubbles) and interception by the X-ray beam can then be varied to provide time resolution. In this case the problem of radiation damage is overcome as well.

When combining techniques one has to be aware of the fact that there might be differences in sensitivity for certain aspects of the experiments. For example, in an extended debate on polymer crystallization that raged roughly between 1998 and 2005, a proposal was made that the distinction between a nucleation and growth crystallization model, and a spinodal-like phase-separation model that preceded crystallization, could be made by the sequential occurrence of scattering and diffraction intensity in the SAXS and WAXS regimes (Terrill *et al.*, 1998 ▶; Strobl, 2000 ▶; Wang *et al.*, 2000 ▶; Olmsted *et al.*, 1998 ▶). The issue that was overlooked was that SAXS would intrinsically be much more sensitive to changes. This is due to the fact that only electron-density differences between different regions need to be detected, independent of whether or not one of these regions is crystalline. On the other hand, WAXS requires a minimum crystalline region size in which the crystalline order is high enough for the effects of Scherrer and disorder peak broadening to be overcome, producing a diffraction peak which can be detected over the statistical background. Thus, one of the phases could be (poorly) crystalline but escape detection by WAXS, whilst the SAXS data would show the existence of a phase-separated sample. Ultimately, this was a case where the quality of the different detectors and the intrinsic sensitivity of the technique, and not the physics of the process, played major roles. In such cases one must either revert to other techniques or use further evidence contained in the X-ray scattering data, such as analysis of the crystallization kinetics (Terrill *et al.*, 1998 ▶; Heeley *et al.*, 2003 ▶), instead of taking a simplified approach looking purely at the order in which events become discernible.

Another example where caution must be exercised is the combination of SAXS with thermal methods. Since the introduction of online differential scanning calorimetry (DSC) (Russell & Koberstein, 1985 ▶; Bras, Derbyshire, Devine *et al.*, 1995 ▶; Kellens *et al.*, 1991 ▶), it has been possible to correlate the phase-transition thermodynamics with structural changes. When a 2 mm thick sample of high-density polyethylene is placed in a beam of 10 keV and 10^12^ photons s^−1^ with a 300 µm diameter, we can expect heating where the beam impinges on the sample, of the order of Δ*T* = 3.4 (K s^−1^), if we assume that no heat exchange will take place between the irradiated area and its surroundings. This does not suggest that any sample will heat up appreciably, since the adiabatic approximation is somewhat rigorous. However, if the thermal sensor of the temperature controller is some distance away from the position where the beam hits the sample, it is not advisable to over-interpret a combination of online DSC and X-ray scattering, since a thermal gradient will almost certainly exist and indeed has been shown experimentally (Warren *et al.*, 2014 ▶). In addition, one should remind oneself of the fact that a beam of 10^12^ photons can turn a polyethylene sample into a cross-linked mess within 60 s.

It is less well known that intense X-ray beams are capable not only of inducing radiation damage but also of promoting particle growth and crystallinity. This is not due to thermal effects but instead to the photoelectric effect that takes place in the sample. The excess of electrons, which in turn can create other radiation products, can influence materials in un­expected ways, even at the length scales probed by SAXS. Even low doses of photoelectrons have been shown to increase the rates of crystallization in glasses (Martis *et al.*, 2011 ▶), to promote the growth of Au particles in glass matrices (Tatchev *et al.*, 2011 ▶) and to induce bundle formation in solutions of macromolecules (Cui *et al.*, 2010 ▶). These effects should be kept in mind when unexpected results are encountered in time-resolved experiments with even moderate flux densities.

## 3. SAXS and WAXS   

When hierarchical length scales are involved, the simplest experimental option is to combine SAXS and WAXS in the conventional transmission mode. Realistically, on most beamlines this can render data over a continuous scattering vector range 3 × 10^−2^ < *q* < 30 (nm^−1^), where *q* is as defined in §1. An example where diffraction and SAXS theory can be applied in a single experiment is the growth of nanocrystals in glasses with a cordierite composition (Bras *et al.*, 2005 ▶, 2009 ▶). After a two-step heat treatment, monodisperse particles were formed. The different stages of particle growth with respect to size and reduction of surface roughness, as revealed by the SAXS data, could be correlated with crystalline lattice deformation and the subsequent relaxation of the system. This was shown to be due to tension in the nanocrystals, caused by volumetric mismatches between the glass matrix and the nanocrystals.

**Figure 1:**
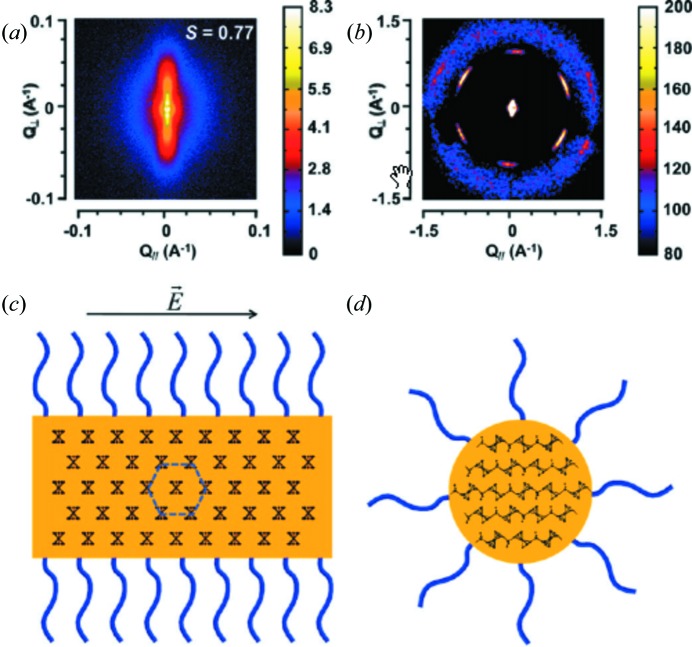
Scattering patterns of block-copolymer PI_550_-b-PFS_50_ in decane solutions. The molecules have self-assembled into rigid cylinders, which are oriented by applying a weak electric field. The combination of (*a*) the SAXS pattern and (*b*) the WAXS pattern is required to elucidate the internal structure of the cylinders in both (*c*) the longitudinal direction and (*d*) the transverse direction, as well as the dimensions of the cylinders and their degree of orientation. Reprinted with permission from Gilroy *et al.* (2011 ▶). Copyright (2011) American Chemical Society.

The rich phase diagram and wide range of relevant length scales found in block copolymer solutions in selective solvents have made the real-time combination of SAXS and WAXS an important part of the experimental toolbox in this research area. As an example, the self-assembly of polyisoprene-block-polyferrocenylsilane, PI_550_-b-PFS_50_, in decane solution can be mentioned (Gilroy *et al.*, 2011 ▶) (Fig. 1 ▶). These materials can self-assemble into rigid rod-like cylinders, after which entropic interactions (Onsager, 1949 ▶) induce a phase transition. By applying a weak electric field an overall orientation can be induced. To elucidate the structure comprehensively, one requires SAXS for the determination of the larger dimensions and the degree of orientation of the cylinders, and WAXS to determine how the molecular packing of the molecules is arranged inside the cylinders.

Another, currently widespread, application is the study of biomineral crystallization. The materials can range from those relevant for human bone formation (Bots *et al.*, 2012 ▶) to sea urchin spikes (Seto *et al.*, 2012 ▶), but the basic principle is the same. Using stop–flow techniques, two solutions are mixed to create a supersaturated solution, from which the crystallization kinetics are followed by SAXS and WAXS. In many cases the existence of an initial amorphous phase is reported, although alternative techniques should be used to confirm whether this is truly amorphous or just poorly ordered. Obviously this methodology is not only appropriate for biological materials but can also be applied to, for example, the formation of technologically relevant nanoparticles (Rath *et al.*, 2014 ▶) in the quest for a better understanding of, and more control over, manufacturing pathways.

**Figure 2:**
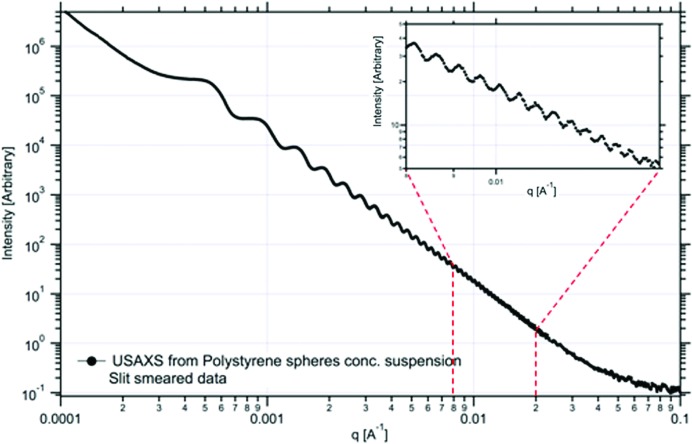
Bonse–Hart ultra small-angle scattering (USAXS) data from monodisperse polystyrene spheres (radius 1 µm). Both the point-to-point and the low-angle resolution over the whole scattering vector range are very high and superior to what is achievable with simple pinhole collimation. Reprinted with permission from Ilavsky *et al.* (2004 ▶). Copyright (2004) American Institute of Physics.

The extension of the observable scattering range towards larger scattering vectors is relatively straightforward, but extension towards lower scattering angles is more problem­atic. A simple increase in the sample-to-detector distance will allow a lower minimum *q* to be observed but, in order to extend the total available scattering vector range, either larger detectors need be used or more separate detection systems have to be installed. Ultimately there is a limit to what is realistically achievable, since in transmission mode the combination of beam size and pixel size will cause very large stretches of *q* space to be mapped onto only a few detector pixels, which severely limits the information content of the experiment. Smaller detector pixels will only have a limited effect in this case. In order to improve this situation and increase the point-to-point resolution, one must revert to Bonse–Hart optics (Ilavsky *et al.*, 2004 ▶). In the early days (Nave *et al.*, 1986 ▶) this method was only suitable for static sample characterization, due to the requirement to rotate the monochromator hardware mechanically, but in recent years this methodology has developed to such a degree that time-resolved experiments (30–60 s per frame) have become feasible. In Fig. 2 ▶, an example is given of the very extended scattering vector range that can be observed with this type of instrument. This figure is not only proof of the improved capabilities of Bonse–Hart cameras, but also shows the extent to which materials scientists and chemists have been able to improve their methodologies to create monodisperse mater­ials. Scattering curves with discernible features over such a wide scattering vector range were the prerogative of biological materials 15–20 years ago, but are now more routinely seen in physical sciences SAXS experiments.

## 4. X-ray scattering and spectroscopic techniques   

**Figure 3:**
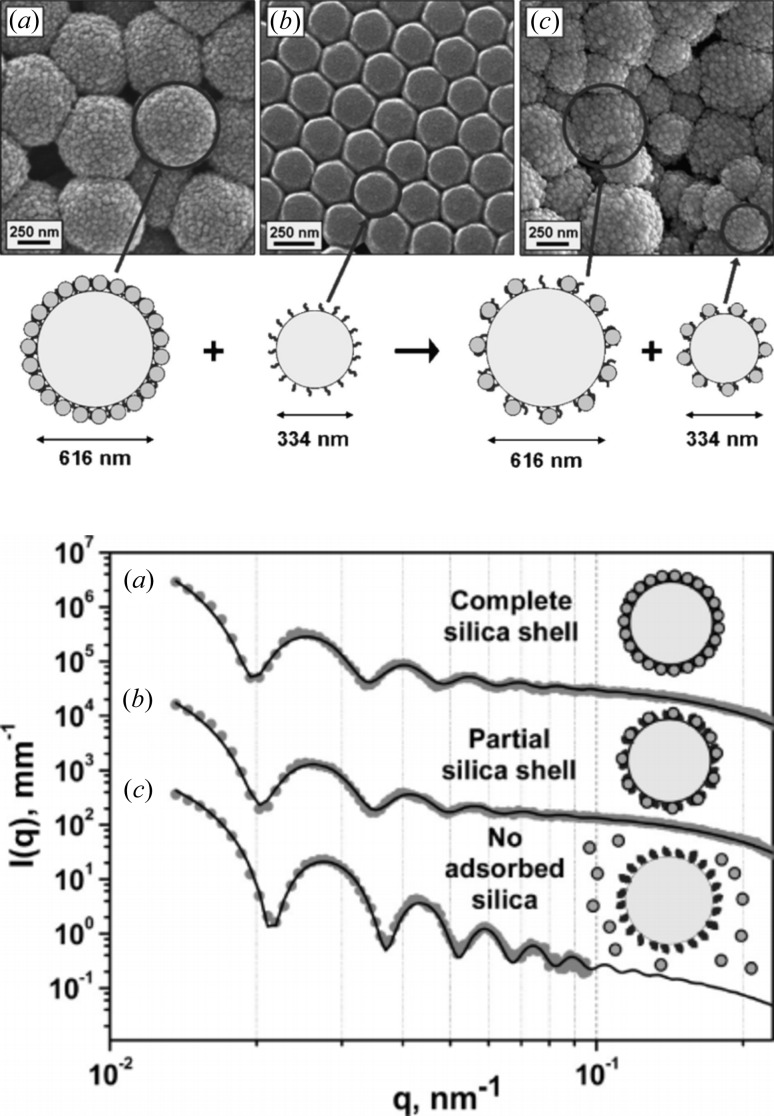
(Top) Electron micrographs and (bottom) scattering curves of silica-based core–shell particles in dilute solution. The availability of the static electron micrographs considerably simplifies the interpretation of the time-resolved scattering data. Reprinted with permission from Mykhaylyk *et al.* (2010 ▶). Copyright (2010) American Chemical Society.

The combination of SAXS with non X-ray based techniques is self-evident, due to the relatively low information content of SAXS data. Having an electron microscope (EM) image of a sample which is to be studied by SAXS is invaluable, since it narrows down the nearly limitless possibilities for data interpretation which are possible when relying only on first principles and SAXS data (Fig. 3 ▶). Another reason is that non-SAXS experts find real-space pictures much easier to comprehend. This can play an important role, especially in an industrial context.

The combination of SAXS with Fourier-transform IR spectroscopy (FT–IR) has been shown to be useful in determining the extent to which chemical reactions have progressed and what this means for structure formation (Bras, Derbyshire, Bogg *et al.*, 1995 ▶). A beautiful example of where FT–IR is used to reveal information not accessible with X-ray methods is in the study of lipid bilayers which form part of the *stratum corneum*, the top protective layer of human skin (Janssens *et al.*, 2012 ▶). This layer has a barrier function which, if impaired, can be a symptom or a cause of skin problems. The lipid bilayers consist of a mixture of ceramides with different chain lengths. The mesoscopic arrangement of these bilayers can be studied by SAXS. The lateral organization of the ceramide head groups inside the layers can be determined to some degree by WAXS, but this technique cannot supply information on the lateral tail organization. By monitoring the CH_2_ symmetric stretching and scissoring vibrations with FT–IR, it is possible to distinguish between the possible (dynamic) tail organizations. Direct correlations between these parameters and the improper skin barrier function suffered by eczema patients were established in this way.

Taking full advantage of time-resolved combinations of SAXS, WAXS and FT–IR has become very much simpler due to advances in FT–IR instrumentation. A good example of this combination is a study of the stress-induced phase transition phenomena of poly(tetramethylene terephthalate) which was uniaxially oriented (Tashiro *et al.*, 2014 ▶). In this study, large-scale lamellar tilts, the structure of the crystalline part and the degree of crystallinity could all be determined simultaneously.

Similar developments have also made the application of online Raman scattering more feasible. Where 16 years ago a combination of SAXS with Raman scattering was still a major instrumentation effort (Bryant *et al.*, 1998 ▶), this has now become very much simpler and one can concentrate on the experiments instead of the instrumentation (Kongmark *et al.*, 2009 ▶). A specific example for the combination of SAXS with Raman scattering can be seen in a study of radiation damage, where the Raman scattering was used to measure the extent of radiation damage in combination with changes in the radius of gyration of proteins in a buffer solution measured by SAXS (Haas *et al.*, 2014 ▶). The same authors also showed that Raman scattering could be used to determine the ratio of bundled to isolated carbon nanotubes. Based purely upon scattering data this would be an ill-posed problem, whereas inclusion of the Raman scattering data allows one to apply the appropriate constraints in the modelling of the SAXS data.

In materials in which amorphous and crystalline regions are interleaved, X-ray scattering tends to be strongly dominated by the crystalline entities. Even though it is feasible to obtain structural information with regard to the amorphous component when the samples are completely in this state, the intensity differences in the scattering/diffraction pattern are such that, in a time-resolved experiment, it is hardly feasible to disentangle the two components and obtain only the pure scattering from the amorphous phase. In semicrystalline polymers, a crystallization from the molten state is simple to follow. As soon as the crystalline domains have a sufficiently large size and ordering, WAXS can be used to follow the crystallization kinetics. In this case, an internally consistent crystallinity scale can be obtained when the degree of crystallinity exceeds 50 vol.%. At that moment the SAXS invariant, 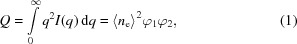
where *n*_e_ is the electron-density difference between the crystalline and amorphous phases and ϕ_*i,j*_ are the volume fractions of the two phases, will reach a maximum. This point can be combined with the zero-crystallinity point in the melt and then correlated with the WAXS peak intensities, which have a nearly linear relation with the degree of crystallinity (Ryan *et al.*, 1995 ▶). However, the X-ray scattering will not yield any serious information about the dynamics in the amorphous fraction which can be slowly transformed into a more crystalline state. In order to gain insights into developments in the amorphous state, one can utilize dielectric spectroscopy (DS) (Sanz *et al.*, 2010 ▶). In this technique the response of the sample to an AC electric field as a function of frequency is measured. The dielectric building blocks of the polymer chain can react to this field and the degrees of freedom in an amorphous fraction can vary. Any potentially existing mesophases with partial ordering will, therefore, have a different response compared with a completely liquid-like state (Ezquerra *et al.*, 2002 ▶; Sics *et al.*, 2000 ▶).

**Figure 4:**
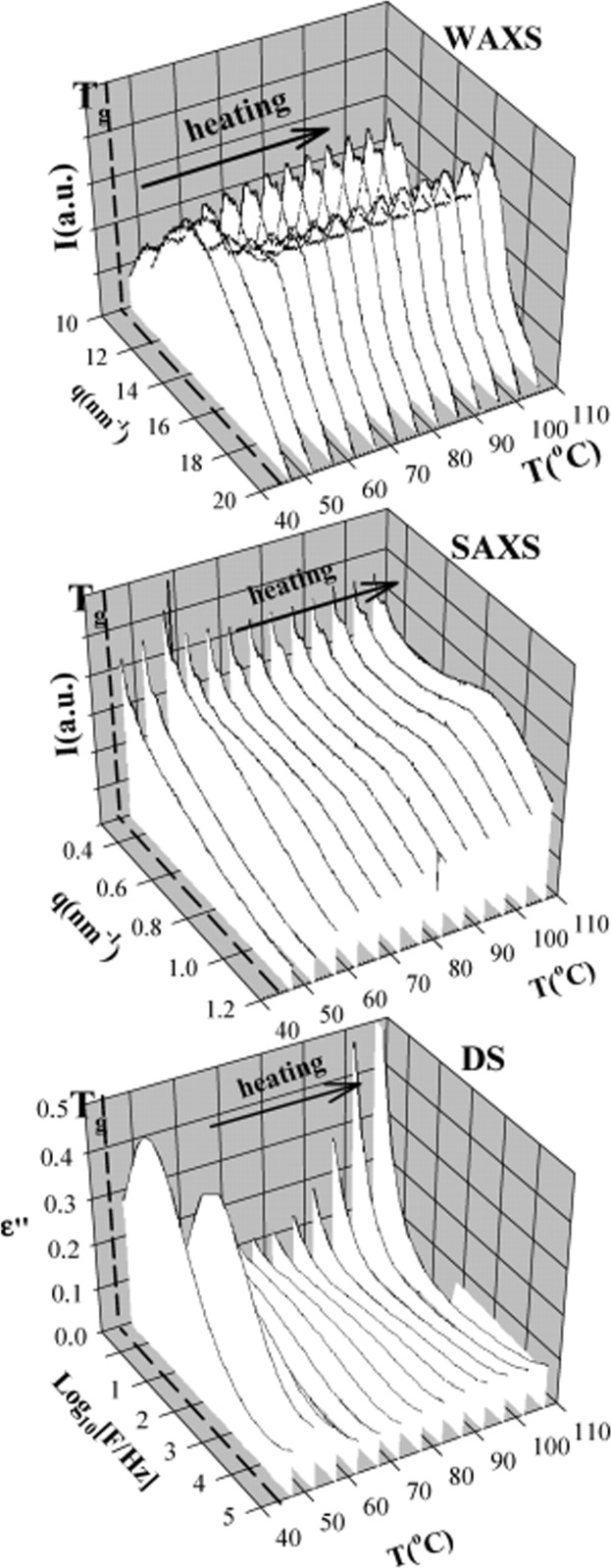
Non-isothermal crystallization of initially amorphous PTT, followed using WAXS (top), SAXS (centre) and DS (bottom), at selected temperatures. WAXS and SAXS intensities are represented as a function of the scattering vector *q*. The bottom panel shows the evolution of the dielectric loss with frequency. Reprinted with permission from Sanz, Nogales *et al.* (2010 ▶). Copyright (2010) American Chemical Society.

An example of this type of experiment is shown in Fig. 4 ▶, where the SAXS, WAXS and DS results from the non-isothermal cold crystallization of poly(trimethylene terephthalate) (PTT) are shown (Sanz, Nogales *et al.*, 2010 ▶). The material is initially amorphous, as showed by the absence of crystalline peaks in the WAXS spectrum. When heated above the glass transition temperature, crystallization starts. The dielectric spectrum initially decreases strongly, indicating that the α-relaxation, associated with segmental motions of the polymer chains, is reduced. This relaxation process only takes place in the amorphous phase and it is therefore not surprising to see the appearance of diffraction peaks in the WAXS and scattering from the crystalline lamellae in the SAXS. A more detailed analysis and temporal correlation of the different phenomena are the basis for a detailed description of the crystallization process.

**Figure 5:**
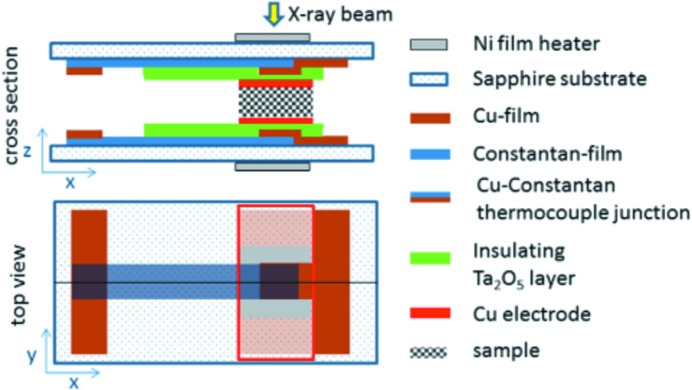
Schematic layout of a cell design suitable for the online combination of dielectric spectroscopy, DSC and SAXS/WAXS. The dimensions of the sample holder are of the order of several millimetres. Due to this low thermal mass, it is also feasible to utilize such designs for very rapid cooling experiments. Reprinted with permission from Wurm *et al.* (2014 ▶). Copyright (2014) Elsevier.

Not only can homogeneous nucleation processes be studied, but also the effects of nucleating agents like multi-walled carbon nanotubes (Wurm *et al.*, 2014 ▶). An ingenious cell, which allows the simultaneous collection of SAXS/WAXS data and DS and DSC traces, was specifically designed for this. Fig. 5 ▶ shows the schematics of the cell design.

A slight note of caution is worthwhile when dealing with very bright X-ray beams and electronic sensors. It is not completely unlikely that photoelectrons generated in the sample might influence the accurate reading of miniature thermocouples or other sensors, even when not placed in the direct beam (Martis *et al.*, 2011 ▶; Chang *et al.*, 2014 ▶).

Thus far, the experiments described have been performed with a fixed X-ray photon energy. However, it is possible to perform X-ray scattering and X-ray spectroscopy (EXAFS or XANES) in a quasi-simultaneous mode (Nikitenko *et al.*, 2008 ▶) with the monochromators now available at most SAXS beamlines. The independent application of these techniques is common practice in, for example, catalysis research, where the catalytic particles are often too small to be investigated by diffraction techniques, and EXAFS/XANES provides information on the direct surroundings and valence states of these particles (Bras & Beale, 2012 ▶). The interaction with, and the larger scale porosity of, the matrix to enable the reactants to reach the catalytic sites is also relevant. This is where diffraction and scattering are relevant (Sankar & Bras, 2009 ▶).

Limitations in time resolution for these experiments are due to the requirement to carry out both a scattering measurement at a fixed energy below the metal absorption edge under investigation and then an EXAFS scan for each time point. The EXAFS scan will be the rate-limiting step here, but time resolutions of 10–60 s per cycle are feasible. Other limitations can exist due to the requirement to obtain the EXAFS data in an energy range determined by the elemental X-ray absorption edge. If this is at energies >15 keV, it will be more difficult to obtain good low-angle resolution data. If the energy is too low, for example around the catalytically important Ti edge of 4.511 keV, the scattering pattern might be so extended that it would be difficult to obtain data over a sufficiently large *q* range. Despite these limitations, there are still sufficient systems where X-ray spectroscopy and X-ray scattering can be usefully combined.

An interesting example of this technique combination is the study of CoAlPO-5 molecular sieves, where Co^2+^ cations are used as both the structure-directing agent and the catalytically active element. Following changes in the local surroundings of the Co^2+^ cation during structure formation is more or less a routine EXAFS investigation. It was assumed that a phase separation occurred somewhere before the onset of crystallization. However, the fact that no relevant changes in the SAXS invariant could be found indicated that crystallization occurred in only one of the phases, and that there was no mass or ionic transport between the phases (Bras *et al.*, 2010 ▶).

**Figure 6:**
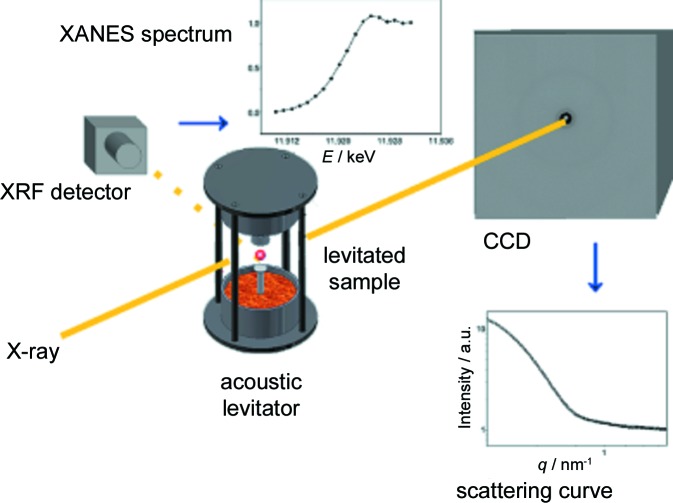
Schematics of an experiment designed to carry out X-ray spectroscopy in combination with X-ray scattering, in order to follow the formation of gold nanoparticles. Influence of cell walls was avoided by using an acoustic levitator (Polte, Kraehnert *et al.*, 2010 ▶).

Following the formation of gold nanoparticles in aqueous solution triggered by the addition of a reducing agent is another example of the combination of XANES and SAXS. For this experiment (Fig. 6 ▶), it was deemed important to eliminate the potential influence of the sample cell walls on the crystallization kinetics. Therefore, a contactless measurement was carried out whereby the sample was levitated by an acoustic levitator (Polte, Ahner *et al.*, 2010 ▶). Obviously such an experimental set-up requires considerable stability of the X-ray beam and the levitation system. In this case, it was possible to show that the formation pathway was not a simple nucleation and growth mechanism but a more complicated path, with both growth *via* monomer addition and ‘non-classical’ crystallization *via* the amalgamation of clusters.

It is clear that the judicious combination of spectroscopic and X-ray scattering techniques considerably increases the information content of experiments. Even though the number of studies utilizing this combination of techniques is increasing, it is somewhat surprising that a survey of the literature reveals relatively few occurrences. The probable reason for this is twofold. Firstly, these are not run-of-the-mill experiments. Beamline users often have to bring part of the equipment themselves, leading to time-consuming installation which, on oversubscribed multi-user facilities, can be a severe drawback. Secondly, there is the unfamiliarity of researchers with the options that are available nowadays.

## 5. BioSAXS and online sample monitoring   

Although this text is mainly focused on materials science, there are some aspects of BioSAXS, or protein solution scattering, which could become relevant for materials science as well. These aspects are mainly to do with online chemistry, online sample quality control and checks for radiation damage.

BioSAXS has been an important component of small-angle scattering since the early days of the technique, and has played an ever-increasing role in structural evaluation in biology since the advent of synchrotron SAXS facilities (Svergun & Koch, 2003 ▶). More recently, a number of robots have been developed to offer high throughput (Blanchet *et al.*, 2012 ▶; Classen *et al.*, 2013 ▶; Nielsen *et al.*, 2012 ▶; Martel *et al.*, 2012 ▶; Pernot *et al.*, 2013 ▶) and improved data collection. To develop biological solution scattering still further, the new third-generation source beamlines have explored chromatography as an adjunct to the scattering technique. Using chromatography to purify samples just prior to injection into a sample measuring module has been around for some time (Mathew *et al.*, 2004 ▶; Watanabe & Inoko, 2009 ▶; Rambo & Tainer, 2010 ▶), but more recently there has been a drive towards using the technique in an analytical sense and for separation (David & Pérez, 2009 ▶; Brookes *et al.*, 2013 ▶). Most popular is size-exclusion chromatography (SEC) which can separate monomers from dimers and multimers, resulting in monodisperse particles for evaluation. This combination has been used to great effect in a number of studies, including protein–ligand binding and following reactions triggered by changes to experimental conditions (Jensen *et al.*, 2010 ▶; Round *et al.*, 2013 ▶). In all cases, the eluted sample is exposed to X-rays immediately after separation. The technique naturally provides a concentration series for these experiments, which previously was effected by a series of independent measurements. Implementation of this kind of experiment is now available at an increasing number of synchrotron facilities worldwide. It has proved to be a good idea to monitor additional biophysical data while carrying out these experiments, to ensure that re-equilibration is not taking place. Recently, several biological SAXS beamlines have added a MALS (multi-angle light scattering) unit to provide additional information and feedback. These biophysical data can then be reviewed against the small-angle scattering information collected, to provide more robust assessment of the molecular profiles obtained.

The kind of online sample quality control described above is implemented in the biological field but is likely to spread to materials science as well. In particular, such developments are gathering pace in catalysis research.

## 6. SAXS and more complicated sample environments   

Although the first SAXS beamline on a synchrotron was set up to perform a rather complicated experiment, *i.e.* to obtain time-resolved diffraction patterns of contracting muscle (Holmes, 1989 ▶), most of the experiments in the early days were simple affairs where static patterns were collected, or samples were subjected to a slow temperature increase. However, even in those early days there were more adventurous experiments undertaken, such as the online polymer fibre-spinning SAXS experiments that were carried out in Hamburg (Cakmak *et al.*, 1993 ▶). Similar experiments were carried out later with the intention of gaining insights into the fundamentals of the very early stages of crystallization. In these experiments, the spinning conditions were chosen such that, on a molecular level, one could consider these to be quiescent conditions (Terrill *et al.*, 1998 ▶). The combination with WAXS was crucial for this type of work. By not only spinning the fibres but also controlling the fibre tension and haul-off speeds, another level of complexity was added (Heeley *et al.*, 2013 ▶). This last step brought these experiments into the realm of industrially relevant research, since now the degree of control over the processing parameters, including thermal profiles, was approaching that used in manufacturing. Similar experiments on film blowing and thermal quenching at industrial speeds have also been reported (Cavallo *et al.*, 2010 ▶). An overview of possibilities in online scattering experiments using relevant industrial polymer-processing methods has appeared recently (Portale *et al.*, 2013 ▶).

**Figure 7:**
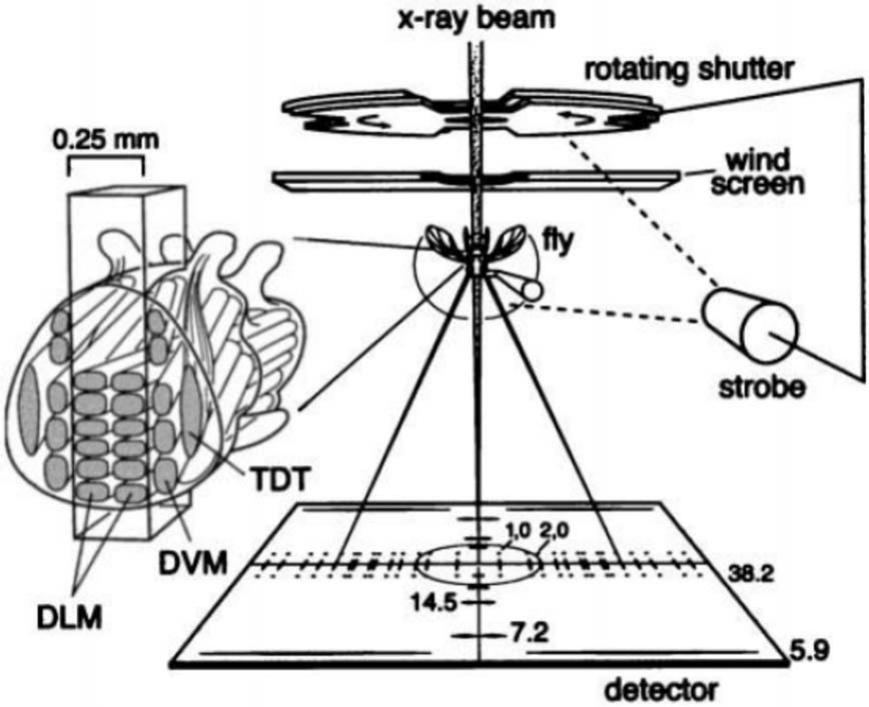
Real-time muscle diffraction experiment on a fruitfly. To keep the wing muscle in the X-ray beam during its power stroke, an optical positioning system had to be constructed that determined the position of the wing and fed this information back to the fly/sample positioning system. Reprinted with permission from Irving & Maughan (2000 ▶). Copyright (2000) Elsevier.

Small-angle fibre diffraction studies of contracting muscle were one of the driving forces in both synchrotron X-ray scattering beamlines and initial detector developments. In view of the relatively small number of groups that have occupied themselves with these studies this is quite remarkable. Apart from pure protein solution scattering experiments, the distinction between biologically relevant studies and materials science tends to become somewhat vague. The many stop–flow experiments on CaCO_3_ crystallization, templated growth, lipid vesicles, silk *etc.* are fully in line with the present paradigm of ‘learning from Nature’. An experiment that has remained firmly inside the realm of biology, and which also shows what experimental ingenuity can do in combination with the present capacities of synchrotron beamlines, is a study of the contraction of *Drosophila* (fruit fly) muscles (Irving & Maughan, 2000 ▶) (Fig. 7 ▶). The experimenters succeeded in keeping the wing muscles from a live *Drosophila* in the X-ray beam whilst the insect was beating its wings with a frequency of around 200 Hz. A complicated optical system using strobo­scopic illumination was designed to determine the wing position in time. This signal was then fed back to a positioning device that can be described as a loudspeaker magnet coil driven by a tone generator.

A muscle-inspired synthetic system based upon a triblock copolymer with hydrophobic end-blocks of poly(methyl methacrylate) and a mid-block of poly(methacrylic) acid, which exhibits a coil–globule transition as a function of pH, has been set up as a free-running motor system by applying a periodically changing pH. The idea was also to show that the cycling in length of the block copolymer reaction could be repeated *ad infinitum* without affecting the structure of the polymer. To achieve, this an oscillating Belousov–Zhabotinsky chemical reaction was used and the chemical ‘factory’ required for this was created *in situ*. To keep track of how much force the sample was generating and to ensure that there was no decay in this, a scanning tunnelling electron microscope tip was attached to one end of the sample. By shining a laser on the tip and measuring the displacement of the reflected laser beam, the deflection, and thus the generated force, could be measured (Howse *et al.*, 2006 ▶).

The above two examples highlight some of the most challenging experiments known to the authors. However, it is clear that not every experiment requires a very complicated setup. To determine the phase diagram of a block copolymer or liposome solution, a simple heating stage and a night of beamtime might be sufficient. What it is important to realise is that such complicated experiments are feasible, and it only requires the will to do them and the perspiration of beamline users and staff to make them a reality.

## 7. SAXS and extreme conditions   

Sometimes the variation of a single parameter, but then *in extremis*, is required. Most synchrotron laboratories have several beamlines and organization units dealing with extreme conditions. The parameters that are most often classified as ‘extreme’ in this context are temperature and pressure. The application of the most extreme conditions is not employed very often in SAXS. This is most likely due to the fact that moderate temperatures are already sufficient to modify the long-range structures that can be studied with SAXS, and extremes of temperature and pressure have a more profound effect on the shorter atomic or molecular length scales.

Alongside temperature, moderate hydrostatic pressure has frequently been used as a variable for studying the kinetics and phase behaviour of systems. High pressure is often used where a clean transition is required to enable the study of kinetics during a phase change. It is widely applicable in the fields where SAXS is relevant. High-pressure cells for this work have been developed by a number of groups (Ando, Chenevier *et al.*, 2008 ▶; Duesing *et al.*, 1996 ▶; Kato & Fujisawa, 1998 ▶; Krywka *et al.*, 2008 ▶; Steinhart *et al.*, 1999 ▶; Brooks *et al.*, 2010 ▶) covering experiments up to 1 GPa. All of the cells referenced above offer fine pressure control essential for the work they carry out, and have a pressure network which allows *P*-jump type experiments. The cells share common features, including custom PTFE (polytetrafluoroethylene) spring seals for work above 0.4 GPa. Recent improvements to cell design have included a dedicated sample-loading port which allows the windows, often diamond, to be left in place during experiments, thus alleviating the problems of background subtraction which were evident in previous designs. Scientifically, the cells have been used to study the distinction between pressure and thermal or chemical denaturation in biological proteins (Ando, Barstow *et al.*, 2008 ▶; Schroer *et al.*, 2010 ▶; Ortore *et al.*, 2009 ▶), how pressure can affect phase transitions in a range of exotic lipid phases (Brooks *et al.*, 2011 ▶; Tang *et al.*, 2012 ▶; Winter & Jeworrek, 2009 ▶), and polymer systems. More recently, diamond anvil cells (DAC), which give access to significantly higher pressures than those described above and which exploit the microfocus capability of many modern low-divergence SAXS beamlines, have been employed in SAXS measurements of systems as wide ranging as starch granules (Gebhardt *et al.*, 2007 ▶) and nanoparticles, where for example a DAC has been used to study the internal composition of hollow γ-Fe_3_O_4_ nanoparticles (Podsiadlo *et al.*, 2013 ▶, 2011 ▶).

High-temperature experiments have been reported on soot formation in gas flames (Hessler *et al.*, 2001 ▶). The required temperature of these experiments is high by definition (>1200 K) and this causes all kinds of problems with background subtractions, since the flames that will produce soot have a different temperature than the soot-less flames. However, some evidence has been found for a multi-step growth process (Gardner *et al.*, 2005 ▶; di Stasio *et al.*, 2006 ▶), although most of the time one has to revert to analysis in the fractal aggregate framework. A rare application of very high temperatures in SAXS is a study of liquid–liquid phase separations in yttrium oxide–aluminium oxide melts (Greaves *et al.*, 2008 ▶). To avoid the potential influence of the sample window material, let alone finding a material that was X-ray transparent, could withstand the high temperatures and was also chemically inert, it was decided to use aerodynamic levitation and laser heating. These experiments were carried out at a temperature of around 1800 K.

Strong superconducting magnetic fields, in the range of 4–10 T, have been used for the online alignment of fibrous biological materials, to induce orientation in order to perform fibre diffraction (Bras *et al.*, 1998 ▶) or even time-resolved re-orientation experiments on liquid crystalline materials (McCulloch *et al.*, 2011 ▶; Brimicombe *et al.*, 2009 ▶). The required superconducting split-coil magnets impose either a technical or a financial limit in increasing the field for a system with a wide bore at room-temperature. Pulsed field magnets up to 30 T have been introduced on synchrotron beamlines, but the pulse duration of 10 ms is too short for controlled experiments on longer-chain molecules (Frings *et al.*, 2006 ▶). Also, the magnetic fields that can be used on current beamlines are still far removed from the extreme magnetic fields of 40 T (static) and 100 T (pulsed) that can be generated in specialized laboratories.

Although the application of a single ‘extreme’ parameter to the sample is not one of the mainstream applications of X-ray scattering, the combination of a variation in pressure, temperature and deformation is quite common and it can justifiably be called an extreme environment.

**Figure 8:**
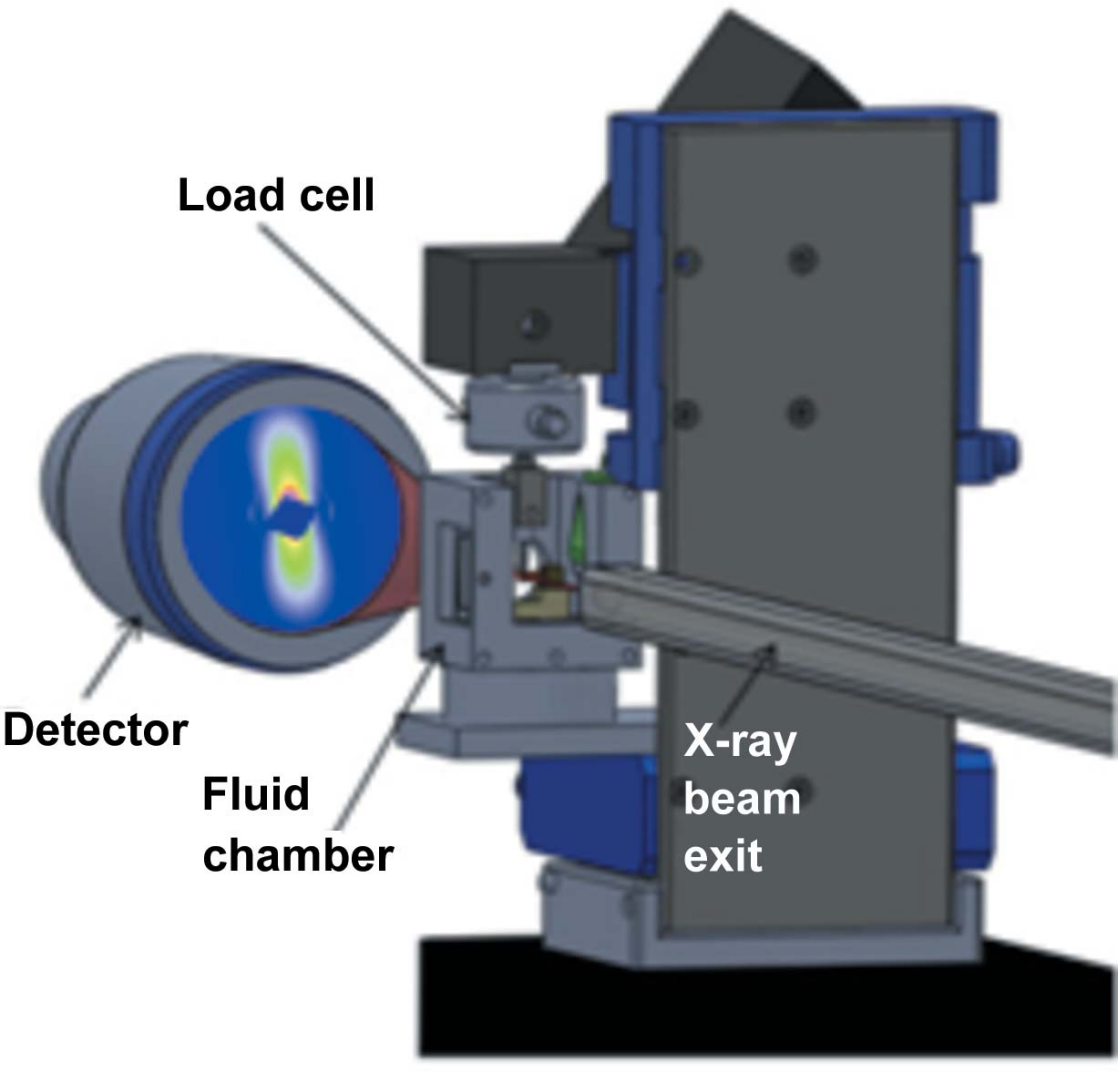
The experimental setup for *in situ* micromechanical (cantilever bending) testing with microfocus SAXS at the I22 beamline, Diamond Light Source, UK. The test specimen was immersed in the fluid chamber by securing inside the metal rig.

One combination of sample environment plus scattering that has been used for some considerable time is extension, be that by stretching the sample or shearing it. Stretching samples can be considered to have started with the muscle experiments described earlier, but were taken up by materials scientists to look at stress-induced crystallization of polymers (Hamley *et al.*, 1998 ▶). More recently, experiments have focused on understanding deformation (Hughes *et al.*, 1999 ▶; Gurun *et al.*, 2009 ▶) and accelerated ageing of potentially exciting new polymer applications in hip implants (Collins *et al.*, 2013 ▶) and artificial heart valves (Stasiak *et al.*, 2011 ▶). This type of experiment also has application in biomaterials, where experiments have most recently been carried out to understand bone deformation (Karunaratne, Esapa, Hiller, Terrill *et al.*, 2012 ▶; Karunaratne, Esapa, Hiller, Boyde *et al.*, 2012 ▶; Karunaratne *et al.*, 2013 ▶). These experiments carried the added challenge of keeping the biological samples in an environmental chamber containing a phosphate-buffered saline solution to mimic physiological conditions. The micromechanical tester (Fig. 8 ▶) has also been used to investigate the axial properties of carbon nanotubes (CNT) (Vilatela *et al.*, 2011 ▶), and to study the structure of and stress transfer in CNT fibres produced by direct spinning from the gas phase during CNT growth.

Coupling rheology with small-angle scattering is a natural extension of the sample environment portfolio already described, where many of the soft-matter systems can be functionally changed by the application of shear. The technique has been applied to a number of areas, including food products (MacMillan *et al.*, 2002 ▶), polymers and block co­polymers (Mykhaylyk *et al.*, 2010 ▶; Mattoussi *et al.*, 1996 ▶), and gels (Crawford *et al.*, 2012 ▶; Newby *et al.*, 2008 ▶). It should be noted that, for practical reasons, the rheological geometries for this type of experiment are limited to a subset of what is available in a stand-alone rheological experiment.

## 8. Advances in time-resolved neutron scattering   

**Figure 9:**
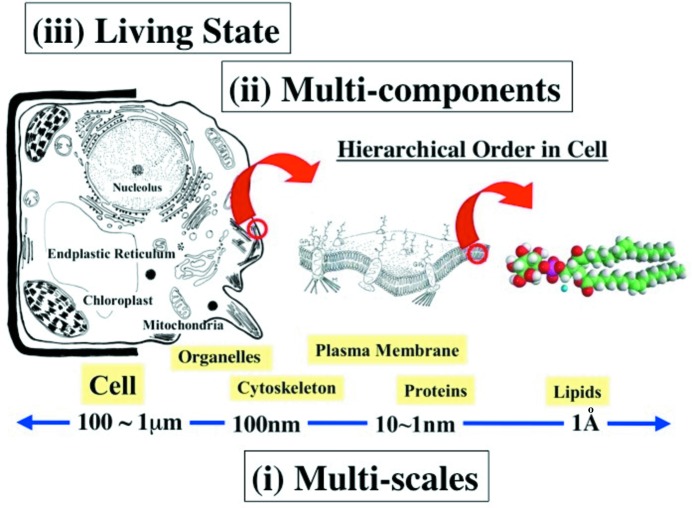
Schematic diagram of hierarchical structures in a cell, composed of multi-component molecules under a non-equilibrium living condition.

A hierarchy of scales is one of the most distinct features in nature. This is shown schematically in Fig. 9 ▶. Plant or animal cells, the size of which ranges from 100 mm down to a few micrometres, contain organelles of size 1 mm–100 nm. The cell and organelles are enclosed by a plasma membrane composed of lipids, the size of which is about 1 nm. In the membrane and cytoplasm there are a huge number of proteins acting as channels, enzymes or cytoskeleton, among others. The important keywords to describe these are: (i) multi-scale systems, (ii) multi-component systems, and (iii) the living state. How does small-angle neutron scattering (SANS) contribute? In order to address the requirements, conventional SANS, which was initiated in the 1970s, has recently been reinforced by coupling with simultaneous functions, as described below for the case of SANS-J-II at JRR-3 Tokai, Japan.

### 8.1. Multi-scale systems   

By employing focusing lenses of biconcave MgF_2_ crystals, or of a Halbach-type sextupole permanent magnetic lens and a high-resolution photomultiplier, the minimum accessible magnitude of the scattering vector *q*_min_ was improved from 3 × 10^−3^ Å^−1^ to an ultra small-angle neutron scattering (USANS) of 3 × 10^−4^ Å^−1^ (Koizumi *et al.*, 2007 ▶). Compared with a Bonse–Hart double-crystal method, which uses a line-focused beam with a smearing effect, the additional advantages of focusing USANS are efficient detection of anisotropic USANS with an area detector, or a gain in neutron flux in the conventional *q* range. By installing a second high-angle area detector, the accessible higher *q* was extended up to 2 Å^−1^. To discriminate incoherent scattering from hydrogen, polarization analysis with a supermirror spin analyser is available on the high-angle detector. After reconstruction, coverage of four orders of magnitude from 10^−4^ to 2.0 Å^−1^ on the same spectro­meter was successfully achieved. If a Bonse–Hart double-crystal USANS spectrometer with grooved perfect crystals and thermal neutrons is used, it is possible to cover a fifth order of magnitude, giving a total *q* range of 10^−5^ to 2.0 Å^−1^. Such a wide *q* range proves useful in the investigation of structures of various sizes that are encountered in biological (Masui *et al.*, 2010 ▶; Koizumi *et al.*, 2008 ▶) or non-biological systems (Koga *et al.*, 2008 ▶; Yamaguchi *et al.*, 2008 ▶). In order to reach macroscopic length scales, a neutron radiography (NR) apparatus, composed of a scintillator, optical mirrors and a CCD camera, was installed at the sample position of SANS-J-II (Iwase *et al.*, 2009 ▶). The new method succeeded in visualizing the water generated in an operating polymer electrolyte fuel cell; NR detected bulk water in a gas diffusion layer and a flow field, whereas SANS quantitatively determined water in a membrane electrode assembly.

### 8.2. Multi-component systems   

For the structural study of multi-component and/or multi-phase systems, a contrast-variation method is essential for determining partial scattering functions or cross-correlations of the components and/or phases. In addition to conventional deuterium labelling for SANS, the scattering length density can be controlled by proton spin polarization. Recently, the novel technique of dynamic nuclear polarization (DNP) has been introduced, which transfers spin polarization from electrons to nuclei, thereby aligning the nuclear spins to the same extent that the electron spins are aligned (Abragam, 1961 ▶). It is accomplished by a DNP target detecting SANS and simultaneously monitoring proton spin polarization by NMR under a condition of low temperature (around 1 K) and high magnetic field (about 5 T) with fine homogeneity. At an early stage of the study of spin contrast variation by SANS (Stuhrmann *et al.*, 1986 ▶), a massive target was prepared and the technique applied to ribosomal protein complexes in solution. The experiment was successful in identifying the microstructure of RNA in the complexes, but with a heavy load and cost. Recently, a compact DNP target was designed, aiming at relatively moderate temperatures above 1 K, which implies imperfect polarization, achieving about 50% (van den Brandt *et al.*, 1995 ▶). A compact DNP target is useful for soft-matter scientists to investigate inhomogeneous dynamic nuclear polarization of protons, coupled with structural analysis, in a lamella-forming diblock copolymer (Noda *et al.*, 2011 ▶).

### 8.3. The living state   

Polymerization solutions are models of living systems, in that the species and content of the solutes change as time proceeds. Anionic copolymerization of styrene and isoprene monomers in a dilute solution, with deuterated benzene as the solvent, has been studied by means of combined time-resolved SANS, size-exclusion chromatography, NMR and UV–Vis spectroscopy (Zhao *et al.*, 2010 ▶, 2009 ▶). As a result, structural changes to the growing (‘living’) chains during the polymerization process were observed at three different length scales using the same solution and in a single batch. This enabled the simultaneous exploration of time-related changes in the local structure (living chain ends), the primary structure (propagating chains) and the higher order structure (the star-like local aggregates of living chains). Similar measurements by SANS and simultaneous methods were performed on various living polymerizations (Hashimoto *et al.*, 2006 ▶; Tanaka *et al.*, 2007 ▶; Terashima *et al.*, 2010 ▶; Motokawa *et al.*, 2010 ▶; Iwase *et al.*, 2011 ▶).

## 9. New developments   

Crystallization studies have been an important driving force for technique developments in time-resolved SAXS/WAXS. The technology for constructing sample environments to make such experiments possible, even at microsecond timescales, has been developed. The difference in sensitivity between SAXS and wide-angle diffraction/scattering events with respect to their ability to shed light on the earliest stages of crystallization, mentioned earlier, has evoked two different responses. The first was to develop very high count-rate single photon-counting WAXS detectors that were at least an order of magnitude more sensitive and efficient than the photon-counting detectors used for SAXS experiments. However, this is an approach that will only bring improvements until one reaches the intrinsic limits, dictated by physics, where differences in technique sensitivity become manifest. Beyond that point one has to revert to different approaches. One of these methods is the use of pair distribution function analysis (Tyrsted *et al.*, 2012 ▶). This technique is definitely not new, but the availability of high-intensity high-energy photon beams, in combination with detectors that can collect photons efficiently at high energies, has brought this technique into the realm of time-resolved experiments. It is a very exciting idea that one can probe the nature of very small particles which can be readily observed by SAXS but whose internal structure still remains somewhat elusive. A question that could be resolved by this technique is, ‘Are the plethora of amorphous precursors which have been reported currently being confused with small poorly ordered crystals, whose diffraction peaks are so weak and broadened that they disappear in the experimental background and statistical noise?’

In data analysis great strides can still be made. Protein solution scientists have been well served by data packages that allow them to squeeze the last piece of information out of their low-information-content data sets consisting of the scattering curves of monodisperse proteins. However, when dealing with time-resolved data from polydisperse particles and high matrix backgrounds, most analysis methods are still *ad hoc* and quite often open to debate. Attempts to utilize similar methods to those used for solution scattering for particle growth have been carried out, but the methods remain cumbersome (Shaw *et al.*, 2002 ▶). Unfortunately, none of the major synchrotron radiation centres has set aside the resources to make this a priority, so we remain in the situation where it all depends on inspired individual developers. The situation for non-isotropic scattering systems is even more lamentable.

When dealing with different experimental data sets obtained in a single time-resolved experiment, it is often not difficult to correlate events. One plots the development of a SAXS invariant on the same graph as the variations in the spectroscopic absorption band, takes a ruler and determines what comes first. However, when subtle effects are in play and the data are noisy, as one can expect in a time-resolved experiment, the situation might become more complicated. Fortunately, there have been some interesting recent developments. Several years ago, two-dimensional correlation analysis on SAXS–WAXS data sets was attempted in order to determine the exact correlation between parameters obtained from the different data sets (Smirnova *et al.*, 2011 ▶). More recently, both convex constraint analysis (CCA) and two-dimensional correlation analyses (2DCOS and 2DHCOS) have been introduced to explore the combination of data sets from different techniques (Haas *et al.*, 2014 ▶). These are promising developments and could further increase the benefits that can be obtained from combined experiments.

## 10. Conclusions   

We have attempted to give a flavour of what is currently feasible in combining techniques with small- and wide-angle scattering. This is by no means an exhaustive review but rather an attempt to make the case; if one thinks that a combination of techniques might deliver results that would otherwise not be available, one should not be afraid to try. On modern SAXS beamlines many things are possible. It might require some hard work and collaboration with the beamline scientists, but that should also be part of the fun of experimenting!
